# Loneliness as Cause

**DOI:** 10.1007/s11245-023-09933-2

**Published:** 2023-06-01

**Authors:** Elena Popa

**Affiliations:** Interdisciplinary Centre for Ethics, Jagiellonian University, Ul. Grodzka 52, 31-044 Kraków, Poland

**Keywords:** Loneliness, Public health, Causality, Interventionism, Mechanisms, Dispositions, Biopsychosocial model

## Abstract

While loneliness has been linked to various mental and physical health problems, the sense in which loneliness is a cause of these conditions has so far attracted little philosophical attention. This paper aims to fill this gap by analyzing research on health effects of loneliness and therapeutic interventions through current approaches to causality. To deal with the problem of causality between psychological, social, and biological variables, the paper endorses a biopsychosocial model of health and disease. I will investigate how three main approaches to causality used in psychiatry and public health apply to loneliness: interventionism, mechanisms, and dispositional theories. Interventionism can specify whether loneliness causes specific effects, or whether a treatment works, incorporating results from randomized controlled trials. Mechanisms help explain how loneliness brings about negative health effects, spelling out psychological processes involved in lonely social cognition. Dispositional approaches help stress particular features of loneliness connected to negative social interactions, such as defensiveness. I will conclude by showing that previous research alongside emerging approaches to health effects of loneliness lend themselves to analysis in terms of the causal models under discussion.

## Introduction

Within health contexts loneliness is increasingly linked to negative impacts on both physical and mental wellbeing. Research in public health has referred to a crisis of loneliness, with additional questions emerging in relation to the COVID-19 pandemic (Holt-Lunstad 2017; Miller 2020). Psychological investigations of loneliness have shed light on pathways through which it affects psychological and physiological processes (Cacioppo and Patrick 2008; Hawkley and Cacioppo 2010). While talk about particular health effects of loneliness presupposes a causal link between loneliness and various health outcomes, the sense in which loneliness causes these problems has not been subject to philosophical analysis thus far. Understanding this causal relation is philosophically interesting because it connects loneliness defined as a subjective feeling with measurable psychological and physiological processes. This paper will discuss loneliness from the perspective of current approaches to causality. As causal generalizations involving loneliness and previous investigations of causality in psychiatry both look for causal links across different levels and domains (i.e., social, psychological, and biological), I will draw especially on work in relation to psychiatry.

I will first review philosophical and psychological work on loneliness in order to specify a concept of loneliness suitable for causal claims within public health (Sect. 1). Subsequently, I will introduce the main approaches to causality applied to psychiatry and public health (Sect. 2). I will then employ these accounts to explain how loneliness can be counted as a cause within a broadly biopsychosocial understanding of health and illness. My analysis will draw on current work on health effects of loneliness and therapeutic interventions, as well as emerging lines of research (Sect. 3).

## Loneliness, Causal Claims, and the Biopsychosocial Model

One challenge that arises when discussing how loneliness brings about health effects is specifying what loneliness is taken to be in the given context. Psychological literature has defined loneliness as a subjective feeling of lacking social connections. This is to be distinguished from social isolation, which amounts to the objective lack of the said connections (Cacioppo and Patrick 2008, p. 5). Nevertheless, philosophical work suggests that loneliness may be more complex. Below, I introduce the relevant philosophical approaches, focusing on insights relevant to the medical context.

As my interest lies chiefly in public health, I will refer to loneliness in the specific sense of chronic loneliness—i.e., as experienced over a long period of time—which can bring about various health conditions. I take it to be a subjective feeling, while at the same time acknowledging the more refined picture provided by philosophical accounts. Particularly, Seemann (2022) singles out features of loneliness captured by three types of views:

- *Objectivism* loneliness is defined in relation to an intentional object—social isolation (Cacioppo and Patrick 2008), or the absence of specific social goods (Roberts and Krueger 2021).
- *Subjectivism* loneliness is experienced as a mood, which shapes one’s understanding of one’s environment; this fits cases of chronic loneliness.
- *Relational views* loneliness is defined as ‘the result of a complex relation between a lack of opportunities for interaction and the narratives that shape one’s self-understanding as being disjointed from one’s environment’ (Seemann 2022, p. 9).

Without taking a stance on the views above, my discussion will touch upon features of all three. When investigating models of how loneliness affects health, social isolation is counted as a cause related to the social environment. Similarly, the way loneliness shapes social cognition will be considered in the context of therapies addressing loneliness and mechanisms through which they work. Lastly, relational aspects are important for highlighting how changes in self-understanding can alter one’s attitude to perceived loneliness, or prompt action toward change.

It is also important to disentangle loneliness construed normatively, as a feeling that may be harmful to the person experiencing it, from experiences of loneliness everyone may go through at times. Particularly relevant in this sense is feminist research criticizing framings of ‘loneliness epidemics’ as failures of individual responsibility for their neglect of political aspects (Wilkinson 2022). I hope that my use of a model of health taking social context into account for discussing loneliness can help address these worries, at least in part, by stressing that loneliness need not come down to individual responsibility. Relational views can help in this sense.

Moving on to the question of causality, examples of causal claims featuring loneliness can refer to both mental and physical health, according to relevant research (e.g., Hawkley and Cacioppo 2010):

- Loneliness causes depressive symptoms.
- Loneliness causes higher rates of cardiovascular mortality.

Establishing how such claims illustrate causality raises the question how a subjective feeling can cause mental and/or biological phenomena. Furthermore, as discussed above, the social background is also relevant for the experience of chronic loneliness. To put this into broader context, causal claims such as those above raise similar questions as claims featuring social causes, such as the following:

- Poverty causes depressive symptoms among women (e.g., Belle 1990).

Medicine has been criticized for neglecting social or structural causes, and predominately focusing on instances of biological causation, such as genetic predispositions, neural processes etc. A recent illustration of this issue regarding loneliness in particular is the neglect of health effects of loneliness in the early response to the COVID-19 pandemic (Popa 2021). The biopsychosocial model, introduced by Engel (1977), can help overcome such shortcomings, but it did not become part of mainstream medicine due to its lack of specificity, among others. Recent work on the biopsychosocial model has pointed out that causal connections between psychosocial variables and biological ones are part of current medical research, particularly the strand on social determinants of illness (Bolton and Gillett 2019). In the following, I first use the example of stress causing various negative health outcomes discussed by Bolton and Gillett, then argue that an analogous analysis can be conducted for loneliness. At the same time, I will take into account further questions and approaches to causality.

Stress has been defined through uncontrollability, namely the presence of environmental demands that exceed one’s psychological resources (Lazarus 1999; Bolton and Gillett 2019). Causal claims in this sense include stress increasing cardiovascular mortality to a similar extent as other known risk factors, such as smoking (Tawakol et al. 2017). Bolton and Gillett particularly look at the mechanism through which stress affects health, describing it as follows: ‘the psychological sense of agency and action itself are compromised, raising risk for mental health problems, because social task demands are excessive and social resources inadequate, and the consequences of this chronic psychosocial misfortune is top-down dysregulation of critical biological processes raising risk of physical health problems’ (2019, p. 126). This mechanism includes psychological elements (one’s sense of agency) alongside social elements (social expectations and available resources) and biological ones (inflammation resulting from chronic exposure to circumstances beyond one’s control). Mechanistic approaches to causality, to be discussed in the following section, fit this picture particularly well. Bolton and Gillett refer to various approaches to causality, as well as to psychiatry as a field where there is a clear intertwining of psychological, social, and biological factors. Further clarification of how causality is understood here is needed before looking at how loneliness can work as another illustration of causal links between psychological and social variables and health outcomes. Before doing so, I will address two related issues that arise with regard to conceptualizing loneliness as a cause: operationalization, and implications of pluralism, particularly incommensurability.

Regarding operationalization, when attempting to measure loneliness so as to describe it as a causal variable one needs to abstract away its complexity, particularly the relational aspects described above. The need for conceptual clarity and the connection between heterogeneous definitions and dearth of effective approaches to address loneliness has been singled out by McHugh Power et al. (2018). Thus, a challenge can be raised whether operationalizing loneliness for causal generalizations may fail to do justice to its complexity. This issue parallels Longino’s discussion of aggression, particularly measuring it as an individual trait versus a relational property (2001, p. 697). The solution is to operationalize loneliness in different ways, using multiple approaches. Longino’s (2013) notion of causal space enables different approaches to measure the target variable in different ways. In the case of loneliness, while psychological approaches may look at how defensiveness undermines one’s social connections, epidemiological approaches may investigate relevant social circumstances, and qualitative approaches may view loneliness as a response to a social environment that fails to provide social goods the individual requires. These approaches can map onto the concepts of loneliness described above: the psychological one matches the subjective concept, the epidemiological one the objective concept, while the qualitative one connects to relational views. As I will explain in Sect. 4 below, this has implications regarding which causal concepts apply to these approaches and concepts of loneliness.

Opting for pluralism, which aligns with my defense of different approaches to causality in what follows, brings about the question of incommensurability. Looking at research on the causes of suicide, Maung (2020) points out that psychological, epidemiological, biological, and qualitative approaches are incommensurable. One source of incommensurability from Maung’s discussion is relevant for my purposes here, concerning how to represent the sets of factors that constitute the causal space (Maung 2020, p. 7). The second one, regarding defining mental disorder is not relevant here, as loneliness is not viewed necessarily as pathological, but as potentially leading to symptoms that may or may not fit psychiatric diagnoses. Using Longino’s (2013) radical pluralism to address the incommensurability problem amounts to accepting that there are multiple, irreconcilable views and causal stories to tell, each providing partial knowledge but no unified view.

By contrast, Mitchell’s (2009) integrative pluralism leaves the door open for integrating different approaches operating at different levels without seeking to reduce them to one another. Acknowledging the possibility of further development as more empirical and conceptual research is conducted on loneliness, my position is that in the current state there is a case to be made for integrative pluralism. The most important tension appears to be between loneliness as mood and loneliness as relational. Yet, the two can be related in ways analogous to how trauma or stress responses have been discussed: subjective loneliness can stem from constant exposure to adverse social environments or deprivation of social goods. Once it is experienced as mood, it can contribute to being further disconnected from one’s social environment, or to missing social goods. Such reinforcing processes that do not seek to reduce one aspect to the other have been brought forward for spelling out the relation between hypervigilance as a trauma response and as a mood that exacerbates anxiety (e.g., Dalgleish et al. 2001). This is also consistent with the observation of McHugh Power et al. that ‘loneliness arises because of factors at multiple levels of functioning, some of which interact with each other’ (2018, p. 225).

## Causality in Psychiatry and Public Health

Current work on causality in psychiatry follows two accounts that have been applied to various scientific domains: interventionism and mechanisms. More broadly, these two views fit the distinction between difference-making and production accounts (Hall 2004), though this includes other theories too (see Illari and Russo 2014). In this section I will review interventionist and mechanistic approaches to causality in psychiatry, then also look at dispositional theories, which are relevant in this context.

An interventionist take on causality in psychiatry has been brought forward by Kendler and Campbell (2009). Broadly, interventionism holds that two variables in a set are causally connected if the value of the putative effect variable can be changed through interventions on the putative cause variable (Woodward 2005). Interventions are defined in a technical sense, drawing on statistical work on causal inference and the ‘graph surgery’ feature (Pearl 2009). The upshot is that the intervention cuts the links between the variable intervened upon and its causes, thus ruling out potential confounders. Applying this to an example from psychiatry, if a certain drug is said to cause recovery from major depressive disorder, testing it would amount to assessing the difference the drug makes to patients. Setting up a controlled experiment where patients suffering from depression are randomly allocated to two groups, one group receiving the drug and the other a placebo, provides the relevant setting for interventions in Pearl’s and Woodward’s sense. By comparing the recovery rates of the two groups, one can determine the extent to which the drug makes a difference to recovery. In psychiatric context, Kendler and Campbell emphasize two advantages of interventionism (2009, p. 882). The first is the ability of including social, psychological, or economic causes without going into the metaphysical intricacies of causality across domains and levels. Since interventionism requires invariance under interventions, showing that mental health is affected by changes in the respective variables would suffice to specify *whether* a causal connection is present without going into detail about *how* one variable affects the other. In the case of psychiatry this is important because if one were to wait for satisfactory solutions to problems such as mental causation or the mind–body problem, one may indefinitely postpone the search for causal connections. Secondly, interventionism allows one to focus on a particular set of variables in order to infer causally, without running into the problem of considering too many variables. Again, this is important in psychiatry given the complexity of mental disorders and their determinants. Connecting this to the biopsychosocial model, Maung (2021) has pointed out that interventionism can overcome shortcomings within the metaphysical background underlying the account by Bolton and Gillett (2019). Its neutrality with regard to contentious metaphysical issues, such as whether there is normativity in nature, appears to be another benefit of interventionism.

Bolton’s reply to Maung that interventionism is insufficient for capturing causal connections within a biopsychosocial model stresses the importance of mechanistic accounts: ‘using the experimental method [i.e., interventionism, my note] only, we so far have no idea how to theorise the biological, psychological or social––so far we just have variable names that we are saying are of these sorts. This is particularly important in this area, because of the centuries old presumptions of materialism and the consequent problematic status of psychological and social causes’ (Bolton 2021, p. 19). To put it another way, determining *whether* a certain factor causes a specific health outcome is insufficient, one also needs knowledge about *how* the said health outcome comes about.

Unlike interventionist views, mechanistic approaches to causality focus on how causes bring about their effects. Mechanisms are discussed in terms of entities and activities contributing to a causal process by Machamer et al. (2000). Subsequent work has referred to interactions between parts of a complex system (Glennan 1996) or to a structure performing a function (Bechtel and Abrahamsen 2005). Aiming to further clarify the notion of mechanism, Illari and Williamson defend the view that ‘a mechanism for a phenomenon consists of entities and activities organized in such a way that they are responsible for the phenomenon’ (2012, p. 120). Discussing causality in psychiatry in terms of mechanisms, Kendler et al. define mental disorders as mechanistic property clusters, i.e., ‘complex, mutually reinforcing networks of causal mechanisms’ (2011, p. 1143). The authors point out that in the context of psychiatric diagnosis no single level can do justice to the complexity of symptoms and their interactions, holding that ‘information about underlying mechanisms will provide new possibilities for classification, but the large number of potentially overlapping mechanisms may mean that there will be no simple and single mapping from mechanism to diagnosis’ (Kendler et al. 2011, p. 1148). The biopsychosocial model discussed above fits this approach, as it argues for looking beyond biological processes involved in various illnesses, to social and psychological ones. Using the example of stress above, the inflammatory responses that lead to heart disease are reinforced by overly demanding social circumstances and psychological feelings of powerlessness. It should be noted that the causal links here do not only go from specific factors and the illness, e.g., social demands and inflammation, but also between different factors that support the underlying state, e.g., social demands, psychological feelings, and physiological processes. Another important aspect captured by mechanistic property clusters is the spiraling trajectory of many of these processes, leading to progressively worse effects on health. Using the example of depression, symptoms such as rumination and negative self-image can reinforce one another, leading to self-fulfilling prophecies, say, if one ruminates about a particular personal or professional failure, leading to subsequent failures being counted as evidence of one’s perceived self-worth.

In light of recent work on causality, and also bringing public health into the picture, the interventionist and mechanistic approaches can complement one another to make sense of multiple factors in health contexts within a pluralist approach (Russo 2022). This builds upon the causal mosaic approach introduced by Illari and Russo (2014). Briefly put, the causal mosaic view holds that none of the current concepts of causation can on its own account for causality across the wide range of domains and contexts. As such, different causal models and concepts can work at the same time, providing distinct parts of an overall picture with regard to particular problems and research questions. Russo (2022) highlights how the causal mosaic approach, and, more broadly, causal pluralism, can address issues regarding the excessive focus on biological causes, and the neglect social or psychological ones. For instance, the prioritization of randomized controlled trials (RCTs) in evidence-based medicine amounts to the employment of the interventionist concept of causation. By allowing for other concepts of causation, such as the mechanistic one, the range of evidence can be expanded (Russo and Williamson 2007). A relevant example of economic variables bringing about health effects is the use of mechanisms to spell out the putative causal connection between tax elasticity of cigarette consumption and smoking intensity (Maziarz 2021).

Taking a pluralistic perspective also draws attention to other approaches to causality that can be employed in public health contexts, of which the dispositional one is important. Dispositional theories of causation focus on properties of the cause variable that confer it the disposition, or power, or capacity to bring about the effect variable, which becomes manifest under appropriate circumstances. Versions of this approach have been defended by Cartwright (1989) and Mumford and Anjum (2011). Rocca and Anjum (2020) discuss dispositional approaches to causation in the context of evidential pluralism applied to public health. Rocca and Anjum hold that identifying intrinsic properties—which are a central part of dispositional theories—can help tell apart mere correlations from causal connections in health contexts. One example is the use of patient narratives, which can provide relevant causal information. For instance, upon investigating a patient with multiple unresolved symptoms including immune dysfunction and chronic pain, finding out about experiences of trauma and abuse in childhood can help establish a causal connection between stress and the predisposition to experience the said symptoms (Rocca and Anjum 2020, p. 6; Song et al. 2018). Another example is that of case studies, which can reveal how intrinsic properties interact to one another and their manifestations: in-depth knowledge about a case, such as a low-income African-American neighborhood, can reveal how race and class experiences determine exposure to pollution, which further affects health (Rocca and Anjum 2020, p. 7). The examples above not only illustrate how singling out intrinsic properties and their manifestations can assist causal inference in public health, but also point to instances of causation between biological, psychological, and social variables, thus also fitting the biopsychosocial model. One point to note here is that dispositional theories and mechanistic ones can single out similar causal connections, say between low socio-economic status and illness, explaining *how* the variables are connected. Nevertheless, they should be distinguished through the aspects on which they focus. Mechanisms look at how entities and activities which are part of a broader structure come together—e.g., socio-economic status, living conditions, working conditions, access to healthcare, and the particular health outcome. Dispositional accounts look at particular features of, say, belonging to a discriminated or marginalized group and how they predispose one to particular health outcomes—e.g., discrimination diminishing one’s opportunities, thus making one more likely to take work in hazardous conditions.

## Causality and Loneliness

I will now look at how loneliness can be framed as a causal variable according to the approaches to causality discussed above. In order to do so, I will rely on previous research on health effects of loneliness. One point to clarify before proceeding is that, in accordance with available studies on loneliness and health, I will look at both interventions meant to improve health by targeting loneliness, and at models depicting the negative health impact of loneliness. As talk of causality in the health sciences comprises determinants of illness, as well the efficacy of specific treatments, the analysis below will include both aspects. Another clarification concerns approaches to reduce loneliness. Talk of treatments raises the question whether loneliness itself should be viewed as pathological, with particular symptoms warranting a diagnosis. As mentioned above, I do not hold that loneliness in general is something to be avoided, but focus on its chronic instance, which has been connected to various health risks. Even in this case, approaches to reduce loneliness need not be confined to pathological cases only. As I will be discussing psychotherapeutic approaches below, it is worth stressing that these therapies need not be limited to individuals with a psychiatric diagnosis, but can also be used to improve mental health. This point can be extended to loneliness: therapeutic approaches that reduce loneliness can help decrease the risk of connected mental and physical health conditions.

One challenge to examining causal claims regarding the efficacy of various interventions to reduce loneliness are the mixed empirical findings. A meta-analysis by Masi et al. (2011) looking at both physical and mental health effects of loneliness has singled out psychotherapeutic approaches to maladaptive social cognition as the most effective. A more recent study by Ma et al. (2020) focusing only on mental health effects has suggested that the evidence for current interventions is inconclusive, calling, among others, for more theoretical work on defining loneliness, as well as for further study of social and environmental determinants of health. This is in line with the considerations on lack of conceptual clarity and the dearth of effective interventions by McHugh Power et al. (2019). Philosophical work on defining loneliness can help open up new ways of measuring loneliness and designing interventions. For example, Motta (2021) points out that the study of loneliness has focused excessively on social relations and related behaviors, emotions, and thoughts. Motta draws attention to phenomenological investigation, which can point out structural features of the lived experience of loneliness (2021, p. 78). Research by Roberts and Krueger (2021) and Ratcliffe (2022) are examples of phenomenological investigations of loneliness experienced as absence. Roberts and Krueger (2021) refer to absence of social goods, while Ratcliffe (2022) discusses loneliness as exclusion from social situations or not belonging. These conceptualizations of loneliness could contribute to qualitative investigations providing a more in-depth perspective on the experiences of affected individuals, beyond simply reporting feeling lonely. Both access to social goods and exclusion can be connected to specific social circumstances, bringing potential social interventions into light. Taking the above-mentioned caveats about inconclusive empirical findings into account, I will analyze the Masi et al. (2011) study from the perspective of contemporary approaches to causation as an illustration of the pluralist approach I defend. I acknowledge that more refined conceptual analysis may yield different studies and findings that lend themselves to causal analysis along the lines I will sketch out.

Employing the interventionist approach amounts to considering loneliness part of a system of variables, where intervening on the putative cause would yield changes in the effect variables. Of particular relevance here is research on interventions to reduce loneliness. Studies identifying potential treatments from previous qualitative research have emphasized improving social skills, improving social support, increasing opportunities for social interaction, and addressing maladaptive social cognition (Masi et al. 2011, p. 219). A meta-analysis on strategies to address loneliness by Masi et al. suggests that interventions addressing maladaptive social cognition are the most effective. Further research highlights the need for RCTs to establish the causal connection between countering maladaptive social cognition and reducing loneliness (Cacioppo et al. 2015). As discussed above, in an RCT people reporting high degrees of loneliness are randomly distributed to a group receiving the treatment and a control group. In this case, the tested approach involves identifying and challenging automatic negative thoughts about others experienced while one feels lonely through cognitive-behavioral therapy (CBT). The causal link between reducing maladaptive social cognition and reducing loneliness is inferred by seeing whether the participants from the first group report feeling less lonely than those in the control group. Looking at this research shows that treatments reducing loneliness can be investigated through the interventionist framework. Nevertheless, in the light of discussions mentioned above, relying on an interventionist concept only does not fully capture the complexity of loneliness. Qualitative approaches to loneliness in particular do not lend themselves to analyses through an interventionist concept of causation. This is because if loneliness is defined through complex social structures and relations, no ‘surgical’ intervention to disconnect the loneliness variable from its causes in the style of controlled experiments is possible. Furthermore, as critiques of evidence-based medicine and its over-reliance on RCTs have shown, the quality of evidence from approaches such as psychotherapy will not be assessed as very high, since the experiment is not blind (Broadbent 2019, ch. 5). Unlike the case when one group is given a specific drug, and another one a placebo, one cannot use psychotherapy without the patient being aware of it. Thus, while an interventionist concept of causation can help investigate *whether* particular treatments help reduce loneliness, other causal concepts are needed to explain *how* that happens, and to connect to other sources of evidence.

Looking at mechanisms now, it is worth stressing that in their meta-analysis Masi et al. also attempt to explain the effectiveness of treatments which addressed maladaptive social cognition, such as CBT. The explanation refers to earlier work in psychology:

This result is consistent with our model of loneliness as regulatory loop (...), in which lonely individuals have increased sensitivity to and surveillance for social threats, preferentially attend to negative social information (...), remember more of the negative aspects of social events (...), hold more negative social expectations (...), and are more likely to behave in ways that confirm their negative expectations (Masi et al 2011, p. 259).

The presence of a loop is consistent with mechanistic property clusters, as discussed above: as symptoms of mental disorders such as depression reinforce one another, so do mental states associated with feelings of loneliness, bringing about behaviors that disrupt social relations. This mechanism has also been presented as follows:

Filtered through the lens of lonely social cognition, other people may appear more critical, competitive, denigrating, or otherwise unwelcoming. These kinds of interpretations quickly become expectations, as loneliness turns the perfectly normal fear of negative evaluation into a readiness to fend off blows. And then the plot thickens. The fear that can force us into a defensive crouch can also cost us some of our ability to self-regulate. When loneliness is protracted, impaired regulation, combined with distorted social cognition, makes us less likely to acknowledge someone else’s perspective (…) The sad irony is that these poorly regulated behaviors, prompted by fearful sensations, often elicit the very rejection that we all dread the most (Cacioppo and Patrick 2009, pp. 22–23).

**Fig. 1:**
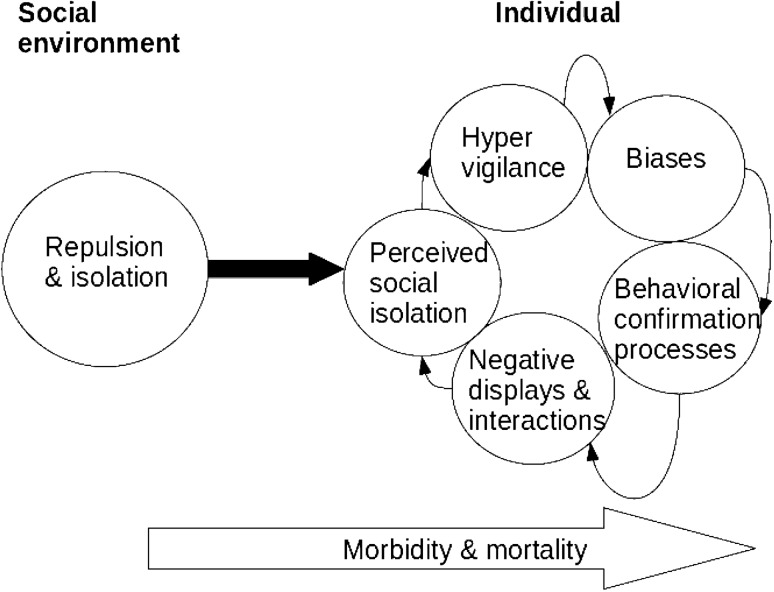
The effects of loneliness on social cognition. Simplified illustration from Cacioppo et al. (2015)

The above illustrates the mutual reinforcement of feelings of loneliness, defensiveness, diminished self-regulation, and negative social interactions. Visual depictions of how loneliness affects social cognition are also consistent with the mechanistic property cluster approach. Figure 1 shows that the manifestations of lonely social cognition such as those described above reinforce one another, forming a loop, and leading to a persistent feeling of loneliness as an underlying state. This process feeds into worse health outcomes over time. The components of a mechanistic property cluster can be singled out as follows:

- *Underlying state* loneliness (as subjective feeling);
- *Causes* e.g., social circumstances, hyper vigilance, biases (confirmation, memory, attention), negative social interactions;
- *Clinical manifestations* e.g., depressive symptoms, high blood pressure contributing to morbidity/mortality rate.

While mentioning contributions to worse health outcomes, the illustration above is missing the physiological processes brought by lonely social cognition, which lead to the deterioration of physical health. This can be complemented by work looking at biological aspects, such as Hawkley and Cacioppo (2010). One challenge here is that most evidence comes from associations, bringing into question whether one can speak of causal connections or only correlations between, say, loneliness and increase in blood pressure. One promising line of research, which can be brought together with the discussion of stress above, is the connection between dysregulation of neuroendocrine processes, which lead to an increase in cortisol levels and inflammation: ‘dysregulation of the HPA [hypothalamic-pituitary adrenocortical] axis contributes to inflammatory processes that play a role in hypertension, atherosclerosis, and coronary heart disease’ (Hawkley and Cacioppo 2010, p. 6). Thus, both stress and loneliness involve top-down dysregulation of biological processes, but do so through different psychological and social mechanisms. While stress involves demands that cannot be met given one’s resources, loneliness involves a disconnection from one’s environment and perceived social threats. A further similarity concerns feelings common in both lonely individuals and individuals experiencing stress, such as lack of control (Adam et al. 2006). This can be related to patterns discussed above, particularly with regard to agency and health. Regarding earlier discussions of pluralism, one thing to note is that mechanistic property clusters are consistent with integrative pluralism insofar as they contain complex mechanisms of biological, psychological, or social nature interacting but without being reducible to one another.

Moving on to dispositional approaches to causation, the models above can also provide insights into intrinsic properties and dispositions related to loneliness. For instance, defensiveness, or the ‘readiness to fend off blows’ can be viewed as a disposition, which manifests itself when one participates in social interactions, leading to negative social displays and the deterioration of relationships. Likewise, dispositional approaches can help single out particular features of loneliness as a mood which shapes one’s perception of reality and their effects. Another important use of the dispositional approach in the context of public health is in identifying patterns of vulnerability. For instance, older individuals are at higher risk of experiencing chronic loneliness. This raises a challenge with respect to whether the susceptibility to chronic loneliness is due to intrinsic properties of old age or retirement, or whether social conditions should also be considered—for instance, if the majority of one’s social interactions involve the workplace. Public health interventions to protect vulnerable groups should take both intrinsic properties and social background into account. As with the other approaches discussed above, the causal connections involve psychological and social processes which generate biological effects.

Having discussed the three approaches to causation in relation to current research on loneliness and health, I will now sketch out further lines of investigation involving loneliness and causality. In describing relational views, according to which loneliness is not only perceived disconnect from one’s social environment, but also involves construing one’s self-understanding through this disconnect, Seemann suggests that narrative therapy can be another way of addressing loneliness (2022, p. 12). Unlike approaches such as CBT, narrative therapy does not place the problem inside the individual, but externalizes it, seeking to enhance possibilities for action through creating new ways of self-understanding (Hutto and Gallagher 2017). Applying this to loneliness, one may shift from understanding oneself as lacking social skills or being incapable of making friends to viewing oneself as being passionate about particular activities, say, reading and talking about books. The latter narrative opens up possibilities such as joining a book club, where one can better connect to people through common interests. Nevertheless, a discussion of causal pathways between narrative therapy and reducing loneliness is hindered by the dearth of empirical evidence. This is partly because initial defenders and practitioners of narrative therapy have resisted empirical approaches during the ‘science wars’.^1^ Further studies can help overcome this, as shown, for instance, in work using benchmarking and clinical significance analyses to show that narrative therapy can be as effective as other psychotherapeutic approaches (Hutto and Gallagher 2017, pp. 162–163; Vromans and Schweitzer 2011). RCTs were conducted for patients with post-traumatic stress disorder (Wilker et al. 2020). Designing RCTs for narrative therapy interventions on loneliness can help single out causal connections in accordance with interventionist models.

At the same time, if narrative therapy is shown to work through RCTs or other methods, further questions about *how* it works are also to be answered. A relevant discussion in this sense concerns how the folk psychological explanations employed in narrative therapy can be spelled out through causal mechanisms from cognitive science (Kirmayer 2015; Hutto and Gallagher 2017). Take, for instance the following causal explanation:

- X refused Y’s help because he felt isolated.
- X’s feeling isolated led to feelings of threat and suspicion towards other people’s motives, which caused him to refuse help.

The former would count as a folk psychological explanation, referring to a particular person, while the latter refers to the mechanism through which lonely social cognition exacerbates loneliness. As pointed out by Hutto and Gallagher (2017), the two need not be in competition. It should also be noted that narrative therapy can be integrated within a broad biopsychosocial model: self-understanding can change through one’s involvement in different activities, including physical ones or by shifting social environments. Thus, causality can involve social, psychological, or biological factors, and causal arrows can go in either direction.

Regarding dispositional accounts of causality, more work is needed to identify particular aspects of narratives that help improve health (Russell et al. 2004). Shedding more light on this can also point to the kinds of narratives patients find helpful and relevant features can be framed in terms of intrinsic properties or dispositions. It is also interesting to note the link between narratives, self-understanding and better health outcomes, and the example of how patient narratives can aid diagnosis discussed above. This suggests that the concepts of causation and causal models investigated here can be used in relation to loneliness across a range of health contexts: diagnosis, treatment, explanation.

## Conclusion

This paper has shown that causal claims involving health effects of loneliness, as well as interventions to reduce loneliness can be spelled out in terms of current accounts of causation. Approaches such as interventionism allow for connections across different levels, while mechanisms can feature psychological, social, and biological processes. Recent work on dispositional theories also singles out connections between psychological variables, such as stress or trauma, and biological ones, such as chronic pain. In line with pluralism, I have argued that using multiple concepts to make sense of causal connections involving loneliness can help not only answer questions whether loneliness has a negative impact on health or whether a certain intervention works, but also how this comes about. I have shown that different concepts of causation are at work within current research on loneliness: in assessing interventions to reduce loneliness through RCTs (interventionism), in spelling out how loneliness disrupts self-regulation increasing the risk of cardiovascular disease (mechanisms), and how the prolonged experience of loneliness or social isolation predisposes one to worse social interactions (dispositional approach). I have also sketched out further lines of research featuring these causal concepts in relation to emerging philosophical and empirical work on narrative therapy.

The discussion here can serve as another example for work on the biopsychosocial model of health and disease. Analogous to stress and its effects on health, loneliness can be viewed as another important determinant of illness that involves biological, psychological, and social aspects. Further empirical research and philosophical clarification on what loneliness amounts to can help single out additional causal pathways, and possibly connect to other causal models.

One limitation is that, given the available empirical evidence, the approaches that have been under the most scrutiny (e.g., CBT) focus on the individual. This can prompt objections from accounts calling for more effort to single out relevant social structures. A way of countering this criticism is to focus on suggested refinements of the concept of loneliness discussed above as well as new areas of research. One such example is the discussion of narrative therapy within a broader context where loneliness can be addressed without necessarily placing responsibility on the individual, allowing for improvement through changes in social environment. While further empirical studies are needed, changing narratives can also prompt social change, for instance, if the elderly are no longer viewed as inactive, or meaningful lives for women are construed beyond the confines of the family. Likewise, I have stressed that dispositional accounts of causation should find ways of bringing together intrinsic properties with features of social environments, which are also relevant for public health. Another answer to this limitation is to extend the analysis to social interventions and policies with regard to loneliness. A relevant starting point here is the review by Bower et al. (2023) on how built environments affect loneliness, calling for investigations of structuration and affordance when researching loneliness from a public health perspective. Once again, this highlights the need for more empirical research on social approaches to reduce loneliness. As with the approaches to causation, one may also hold that having multiple ways of addressing loneliness can help work across different contexts and cases.

## Data Availability

This article employs philosophical analysis of already published empirical studies. As such, it does not produce any new empirical data.

## Footnotes

1 For an overview, see Murdock (2004, ch. 15).
